# The Role of Protein-Ligand Contacts in Allosteric Regulation of the *Escherichia coli* Catabolite Activator Protein*

**DOI:** 10.1074/jbc.M115.669267

**Published:** 2015-07-16

**Authors:** Philip D. Townsend, Thomas L. Rodgers, Laura C. Glover, Heidi J. Korhonen, Shane A. Richards, Lucy J. Colwell, Ehmke Pohl, Mark R. Wilson, David R. W. Hodgson, Tom C. B. McLeish, Martin J. Cann

**Affiliations:** From the ‡ School of Biological and Biomedical Sciences,; the § Biophysical Sciences Institute, and; the Departments of ‖ Chemistry and; §§ Physics, Durham University, Durham DH1 3LE, United Kingdom,; the ¶ School of Chemical Engineering and Analytical Science, University of Manchester, Manchester M13 9PL, United Kingdom,; the ** Department of Chemistry, University of Turku, 20014 Turku, Finland, and; the ‡‡ Department of Chemistry, University of Cambridge, Cambridge CB2 1EW, United Kingdom

**Keywords:** allosteric regulation, biophysics, calorimetry, cyclic AMP (cAMP), nucleoside/nucleotide analogue, protein dynamic

## Abstract

Allostery is a fundamental process by which ligand binding to a protein alters its activity at a distant site. Both experimental and theoretical evidence demonstrate that allostery can be communicated through altered slow relaxation protein dynamics without conformational change. The catabolite activator protein (CAP) of *Escherichia coli* is an exemplar for the analysis of such entropically driven allostery. Negative allostery in CAP occurs between identical cAMP binding sites. Changes to the cAMP-binding pocket can therefore impact the allosteric properties of CAP. Here we demonstrate, through a combination of coarse-grained modeling, isothermal calorimetry, and structural analysis, that decreasing the affinity of CAP for cAMP enhances negative cooperativity through an entropic penalty for ligand binding. The use of variant cAMP ligands indicates the data are not explained by structural heterogeneity between protein mutants. We observe computationally that altered interaction strength between CAP and cAMP variously modifies the change in allosteric cooperativity due to second site CAP mutations. As the degree of correlated motion between the cAMP-contacting site and a second site on CAP increases, there is a tendency for computed double mutations at these sites to drive CAP toward noncooperativity. Naturally occurring pairs of covarying residues in CAP do not display this tendency, suggesting a selection pressure to fine tune allostery on changes to the CAP ligand-binding pocket without a drive to a noncooperative state. In general, we hypothesize an evolutionary selection pressure to retain slow relaxation dynamics-induced allostery in proteins in which evolution of the ligand-binding site is occurring.

## Teaser

**Background:** Protein allostery can be communicated purely through altered entropy.

**Results:** Altered cAMP binding strength in CAP results in changes to entropy-driven allostery.

**Conclusion:** The requirement to maintain allostery constrains evolution of the ligand-binding site in CAP.

**Significance:** Entropy-driven processes can constrain amino acid covariation in evolution.

## Introduction

Allostery (from the Greek *allos stereos* “other solid”) is the regulation of protein function through the binding of an effector molecule at a site remote from the active site and can switch proteins between active and inactive states (1). Allostery is a fundamental mechanism that underpins both normal and pathological cellular processes (2). Our current understanding of the molecular basis of allostery comes from both theoretical and experimental studies and frequently describes the process in terms of protein conformational change (3, 4). A combination of x-ray crystallography and NMR have served as useful tools to permit an analysis of the ligand binding sites, intermolecular interactions, and conformational fluctuations that describe a range of allosteric systems (5–7). It has become clear from such analyses that allosteric connectivity between distinct sites can, in certain cases, be communicated by changes in the protein dynamics of thermal fluctuations alone (8–14). For example, the ensemble allosteric model describes allostery as arising from ligand-derived stabilization of a dynamic ensemble of protein states (15–17).

A theoretical study by Cooper and Dryden (8) laid the theoretical foundations for such dynamic control of allostery. Their statistical thermodynamic formulation indicted that allosteric free energies of the order of several kJ mol^−1^ could be achieved through ligand-induced changes to protein dynamics. This was achieved primarily through changes in entropy arising from the altered amplitudes of protein thermal motions. The effect can occur even in allosteric proteins that do exhibit structural change on binding as demonstrated for the Lac repressor (18). This indicates that evolution has enabled proteins to take functional advantage of not only their mean conformation but also inherent thermal fluctuations about this mean (19–24).

Work in numerous systems suggests that there is no single current predictive framework that describes the contribution of protein dynamics to allosteric control. Allosteric phenomena have been described as a continuum in which different allosteric systems can be artificially ordered according to the extent of the dynamical change present (2). At one extreme are relatively rigid systems such as allostery in hemoglobin through rigid body motions (25, 26), and side chain dynamics in PDZ domains (27). At the other extreme are systems that control allostery arising through protein unfolding and intrinsic disorder, including the TetR repressor DNA-binding domain (28) and the Phd/Doc toxin-antitoxin system (29). Intermediate between these two extremes are structured yet inherently flexible allosteric systems of which the CRP/FNR family transcription factor CAP^2^ of *Escherichia coli* is a well studied exemplar. CAP is a 210-amino acid homodimeric transcription factor that binds cAMP generated by adenylyl cyclase in response to the phosphorylated form of Enzyme IIA^Glc^ (phosphorylated in response to the phosphoenolpyruvate-carbohydrate phosphotransferase system) (30). CAP regulates the transcription of over 100 genes required for the metabolism of diverse carbon sources through its binding to a specific promoter region and recruitment of RNA polymerase (31). Analysis of the two major ligand binding domains of the CAP homodimer demonstrated a homotropic negative allosteric interaction between cAMP binding sites and the absence of structural change within this domain (12). This finding supports the theory that allostery can occur through predominantly entropic processes. Additional studies have demonstrated that conformational entropy via altered backbone and side chain dynamics is also linked to allostery between ligand and DNA binding (14, 32). The demonstration of entropically driven allostery in CAP generated immediate questions about its mechanism. Seven of eight CAP mutants previously examined showed a direct correlation between ΔΔ*G* and the adiabatic compressibility (β_s_°): proteins with a higher β_s_° (reflecting increased structural flexibility in solution) demonstrated enhanced negative cooperativity (33). These experimental findings support the idea that altered correlations in global motion play a role in the regulation of allostery in CAP.

Computational studies support these experimental results and offer some fundamental insights. The normal modes describe the different, globally correlated, harmonic vibrational oscillations in a protein around a mean minimum energy protein structure. An elastic network model (ENM) represents a coarse-grained modeling approach to investigate the role of the modes and their modification in protein function (34–36). ENMs have proven particularly useful in the analyses of slow relaxation dynamics in protein function (37–40). A study of CAP using ENMs demonstrated that negative allostery arises from modulation of the global slow relaxation modes on cAMP binding (41–44). Furthermore, the elucidated theoretical framework permitted the prediction of other “second sites” where modification in local rigidity exerted varying levels of control over allostery between effector and allosteric sites in CAP and the rational modulation of allostery through protein engineering at these control sites (42).

A further question that arises from the use of ENMs in the analysis of allostery in CAP is the relationship between the contacts formed within the binding site itself between CAP and cAMP and their control over allostery. Here we present a combined theoretical and experimental analysis of this question and its relationship to protein evolution. We show that reducing the strength of binding between CAP and cAMP from its wild type value enhances negative cooperativity. Further, reducing the strength of binding between CAP and cAMP in the presence of computed mutations within the protein (here referred to as second site mutations) tends to reduce cooperativity, depending upon the degree of correlated motion between sites. An analysis of naturally occurring CAP variants reveals a subset of residues that possess a mutational covariance with cAMP-contacting sites. These residues from naturally occurring CAP variants form a distinct population that does not show the same pattern of reduced cooperativity when mutated on simulation. This suggests an evolutionary selection pressure to retain fluctuation-induced allostery when evolution of the ligand-binding site is occurring.

## Experimental Procedures

### 

#### 

##### Chemicals

Adeonsine-3′,5′-cyclic monophosphorothioate, Sp-isomer (Sp-cAMPS) and 2′-amino-2′-deoxyadenosine-3′,5′-cyclic monophosphate (2′-NH_2_-cAMP) were purchased from Biolog (Bremen, Germany). 2′-deoxyadenosine 3′,5′-cyclic monophosphate (2′-deoxy-cAMP) was purchased from Sigma-Aldrich. For inosine-3′,5′-cyclic monophosphate, inosine (0.26 g, 1 mmol) was dried over phosphorus pentoxide overnight and dissolved in 5 ml of freshly distilled triethyl phosphate with heating. The solution was cooled to room temperature, and freshly distilled phosporyl chloride (186 μl, 2 mmol) was added. The mixture was stirred for 3 h at 0 °C, added to a stirred solution of 0.08 m potassium hydroxide in H_2_O/acetonitrile (4:6, 120 ml) at 0 °C, and neutralized to pH 7 with 1 m HCl. The organic solvent was removed *in vacuo*, and the residue was extracted with ether (2 × 100 ml). The residue was added to 100 ml of methanol to precipitate insoluble salts, which were removed by filtration. Lyophilization yielded the crude product, which was suspended in 10 ml of H_2_O. Inorganic phosphate was precipitated by adding acetone (2.2 v/v) dropwise while stirring at 0 °C. After 30 min, the mixture was centrifuged, and the liquid and solid were separated. Acetone was removed from the liquid layer by rotary evaporator, and the product in the remaining aqueous layer was purified by DEAE ion exchange chromatography using a linear gradient of 19–200 mm triethylammonium bicarbonate buffer. The fractions containing product were lyophilized to give inosine-3′,5′-cyclic monophosphate as its triethylammonium salt. δ_H_ (400.06 MHz, D_2_O) 8.11 (2H, s), 6.10 (1H, s), 4.42 (1H, dd, J_1_ = 22 Hz, J_2_ = 4 Hz), 4.23 (2H, m); δ_P_ (161.96 MHz, D_2_O) −1.7 (1P, d, J = 21 Hz); ES *m*/*z* 329.0 ([M-H]−).

##### Biochemistry

CAP protein was expressed and purified, and isothermal calorimetry was performed as previously described (42). Raw isothermal calorimetry data were processed by integrating the areas under each peak to generate a binding isotherm and modeled using MicroCal Origin 7 software.

##### Crystal Structure Determinations

CAP crystals were produced at pH 6.5 with 7–10% (w/v) polyethylene glycol 3350 and 15–20% (v/v) 2-methyl-2,4-pentanediol with 2 mm cAMP or Sp-cAMPS in 24-well hanging drop vapor diffusion plates. All diffraction data were collected at the Diamond Light Source beam I-02 and processed using Mosflm (45) and Scala (46). CAP structures were solved using molecular replacement with Phaser (47) using CAP (Protein Data Bank code 1I5Z). Model building and refinement were accomplished iteratively using COOT (48) and Refmac5 (49) in CCP4 (45). Structural and refinement statistics are provided in Table 1. The structures presented here contain one dimer (wild type CAP with Sp-cAMPs in space group P2_1_) and two dimers (wild type CAP with cAMP in space group P1). In all cases, the dimers are symmetric with no significant differences between the two protein chains that form the functional dimer. Superpositions were performed using SUPER in PyMOL. Diffraction data and coordinates have been deposited to the protein database under accession code 4R8H.

**TABLE 1:** **Crystallographic data collection and refinement statistics**

**Data collection**	
X-ray source	I04*^a^*
Wavelength (Å)	0.9687
Space group	*P* 1 2_1_ 1
Cell dimensions	
*a*, *b*, *c* (Å)	45.7, 102.3, 54.3
α, β, γ (°)	90.0, 111.6, 90.0
Resolution (Å)*^b^*	50.5 (1.50–1.46)
*R*_merge_ (%)*^b^*^,^*^c^*	5.5 (64.5)
*I*/σ(*I*)*^b^*	14.7 (2.3)
Completeness (%)*^b^*	99.6 (98.9)
Multiplicity*^b^*	4.5 (4.0)

**Refinement**	
Resolution (Å)	1.46
No. measurements	358,197
No. unique	80,040
*R*_work_/*R*_free_	0.1871/0.2150
No. atoms	
Protein	3126
Ligand/ion	129
Water	255
Average B-factor	30.889
Root mean square deviations	
Bond lengths (Å)	0.0138
Bond angles (°)	1.8058
Protein Data Bank code	4R8H

*^a^* Diamond Light Source.

*^b^* Number in parentheses is for the last shell.

*^c^* *r* = Σ(Abs(*I* − <*I*>)/Σ(*I*)).

##### Coarse-grained Simulations

ENM simulations were performed using the ΔΔPT software package (36, 41). The spring constants were set to a constant value of 1 kcal mol^−1^ Å^−2^ with a cutoff radius of 8 Å, and only the Cα atoms in the protein were considered. The presence of cAMP effector at the binding site was treated by the addition of one node at the mass weighted average coordinate for each ligand atom. Varying the spring constant of any springs attached to a single residue of the protein was used to represent side chain mutations. The allosteric free energy was calculated by summing over the first 100 modes. All ENMs described are calculated from an x-ray structure determination of CAP-cAMP (Protein Data Bank code 1G6N).

##### Identification of Covarying Amino Acids in CAP Variants

An alignment of CAP homologues was constructed using HHblits (50) using a BLAST value of 10E^−20^ as a threshold for homologue inclusion (11,023 sequences). Amino acid covariation was scored using the parameters of a maximum entropy model inferred from the sequence alignment.

##### Spearman's Rank Correlation

A randomization test was performed to determine whether the set of 35 observed covarying amino acid pairings were consistent with all theoretical pairings. First, a Spearman's rank order correlation (SRC) test was performed on the observed data to obtain the SRC coefficient, *r.* 10,000 random pairings of size 35 were drawn from the group of theoretical pairings with replacement, and for each draw a SRC test was performed. A *p* value was estimated by noting the ranking of the observed SRC coefficient among the simulated coefficients.

## Results

**FIGURE 1.**
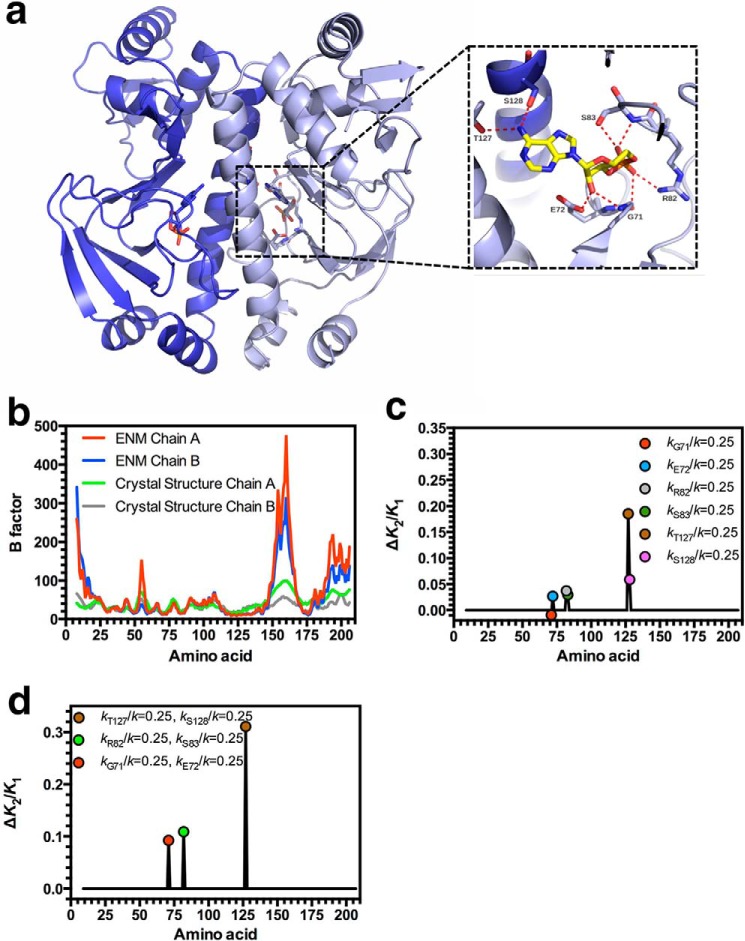
**The influence of CAP-cAMP contacts on allostery in CAP.** *a*, ribbon diagram of the x-ray crystal structure of CAP (Protein Data Bank code 4HZF) showing the secondary and tertiary structures of the CAP homodimer with cAMP bound. The *inset* shows the hydrogen-bonding network at the cAMP binding site in the wild type protein. The structure and *inset* are shown in different orientations for clarity. The labeled amino acids in the *inset* contact cAMP and are analyzed in this study. *b*, B-factor plotted against amino acid number for the crystal structure and the manually curated ENM for chain A and chain B of the CAP crystal structure. *c*, the change in cooperativity (*K*_2_/*K*_1_) that occurs when *k*_R_/*k* is varied at the indicated residue. *d*, the change in cooperativity (*K*_2_/*K*_1_) that occurs when *k*_R_/*k* is varied for pairs of indicated residues.

Negative allosteric cooperativity between cAMP binding sites in CAP occurs as a consequence of the protein-ligand contacts formed. Knowledge of how the interactions between cAMP and CAP contribute to allostery through alteration in global slow relaxation motion can assist in interpreting selection pressures on protein evolution. The x-ray crystal structure for the CAP holoprotein at 1.48 Å resolution revealed the contacts made between CAP and cAMP (Fig. 1*a*). Thr-127 and Ser-128 are hydrogen bond donors to the adenine ring amine group. The main chain nitrogen of Gly-71 and the Glu-72 side chain carboxyl form hydrogen bonds with the 2′-hydroyl group of the ribose, whereas the main chain carbonyl of Gly-71, the Arg-82 side chain, and Ser-83 main chain nitrogen and side chain form hydrogen bonds with the phosphate of cAMP (51–53).

We used ENM level coarse-grained modeling to predict the influence of residues that directly contact cAMP on allostery and subsequently validated these predictions through experimental analysis. Such an analysis of the influence of ligand-protein interactions on allostery is a prerequisite to investigate the evolutionary constraints that arise from this interaction. An ENM was constructed from a high resolution crystal structure by taking the positions of the Cα atoms and connecting all pairs of Cα atoms within a specified cutoff distance of 8 Å using simple harmonic springs with the ΔΔPT software package (41). This process was performed for the three states of CAP-cAMP binding with the relevant ligand included as appropriate. There is no difference in structure between the ENM models representing the apo and single and double cAMP bound states. Similar models have previously demonstrated the entropic contribution to negative cooperativity in CAP despite the thermodynamic contribution of motions of the DNA-binding domain in the protein (42). The ENM was modified to remove springs within the 8 Å cutoff that do not correspond with CAP-cAMP interactions as identified from the x-ray crystal structure (Fig. 1*a*). The binding between CAP and cAMP was therefore created with springs between the six Cα atoms corresponding to the residues that contact cAMP (Gly-71, Glu-72, Arg-82, Ser-83, Thr-127, and Ser-128) and cAMP. Free energies, Δ*G*, were calculated using the full harmonic solution summed over the first 100 normal modes, and the negatively allosteric binding of cAMP to wild type full-length CAP confirmed in the modified ENM by calculating a positive value for ΔΔ*G* = (Δ*G*_holo2_ − Δ*G*_holo1_) − (Δ*G*_holo1_ − Δ*G*_apo_) = 93.5 cal mol^−1^ (equivalent to an allosteric index of *K*_2_/*K*_1_ = 1.17, where *K*_1_ and *K*_2_ represent the dissociation constants for the first and second cAMP binding events).

Experimental B-factors can be used as a reasonable approximation of local motions in solution when static disorder is smaller than dynamic disorder (54). A comparison of the B-factor data for the modified ENM was qualitatively similar to the crystallographic B-factor data except for larger deviations at the unconstrained termini and flexible loop regions (Fig. 1*b*).

The ENM can make qualitative predictions regarding the influence of the altered Cα-cAMP interactions on the allosteric index, through changes to the force constants for springs that make contact between CAP and cAMP. We examined the influence of each identified interaction between CAP and cAMP by introducing appropriate mutations into the ENM. The mutations were modeled by changing the spring constants between the appropriate Cα atom(s) of the ENM and the associated ligand (42). The force constant for each spring within the ENM was assigned a nominal wild type value of *k*_R_/*k* = 1 (*k*_R_/*k*; corresponds to *k*_amino acid number_/standard ENM spring strength). In the case of a mutation, *k*_R_/*k* was reduced to a value of 0.25 to represent the decreased binding strength. The consequent change to *K*_2_/*K*_1_ was calculated to investigate the effect of the simulated mutation on cooperativity (Fig. 1*c*). A reduction in force constant at five of the identified sites increased *K*_2_/*K*_1_ (*i.e.* increases the extent of negative cooperativity), whereas at a single site (*k*_G71_/*k* = 0.25), *K*_2_/*K*_1_ decreased (*i.e.* decreases the magnitude of negative cooperativity). The six identified residues can also be viewed as three interacting pairs (Gly-71/Glu-72, Arg-82/Ser-83, and Thr-127/Ser-128) at three positions on cAMP. We therefore subsequently examined the influence of reducing the force constants for the springs within the ENM in pairs (Fig. 1*d*). The interaction between the two residues in each of the three pairs was nonadditive, and in each case an increase in negative cooperativity was observed.

**FIGURE 2.**
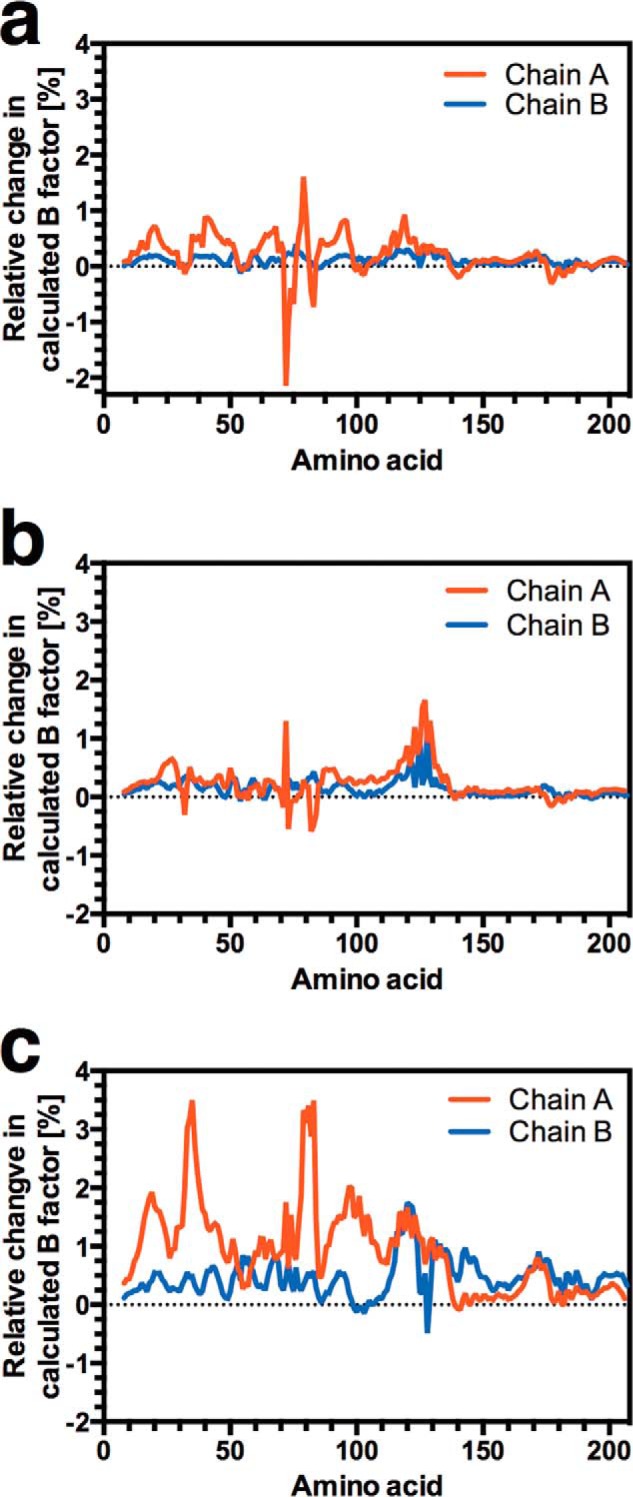
**Mapping local dynamics in CAP.** The effect of mutation of Gly-71/Glu-72 (*a*), Arg-82/Ser-83 (*b*), and Thr-127/Ser-128 (*c*) on local dynamics over the CAP monomer. The chart represents the percentage variation in the calculated B-factor from the wild type curated ENM plotted against amino acid number. The ENMs represent the single ligand-bound state with one molecule of cAMP bound to chain A.

The data indicate that weakened CAP-cAMP interactions in the ENM alters *K*_2_/*K*_1_ through changes to the normal modes and correlated slow relaxation motions. Specifically, we therefore anticipated global changes to Cα flexibility as CAP-cAMP interactions are altered. Modifications to simulated backbone flexibility are present throughout CAP with varying amplitude but generally show loosening of the protein in the *k*_G71_/*k* = 0.25, *k*_E72_/*k* = 0.25 (ENM^G71/E72^; Fig. 2*a*), *k*_R82_/*k* = 0.25, *k*_S83_/*k* = 0.25 (ENM^R82/S83^; Fig. 2*b*), and *k*_T127_/*k* = 0.25, *k*_S183_/*k* = 0.25 (ENM^T127/S128^; Fig. 2*c*) ENMs with a single molecule of cAMP bound to chain A. The percentage change to global backbone flexibility is consistent with changes observed with simulated mutations introduced at the CAP dimer interface and surface loops (42). An increase in *K*_2_/*K*_1_ on reduced CAP-cAMP interaction strength is therefore associated with increased nonlocal backbone motion in chain B, which will bind the second molecule of cAMP. This is consistent with the alteration in the normal modes in CAP as the controlling mechanism for changes in *K*_2_/*K*_1_. The interaction between the enhanced backbone motions following the first cAMP binding event creates an entropic contribution to negative cooperativity in ΔΔ*G* as modeled here (11). We conclude, therefore, that evolution of the ligand-binding site can influence both ligand affinity and allosteric properties. This establishes a likely source of an evolutionary constraint for further investigation.

**FIGURE 3.**
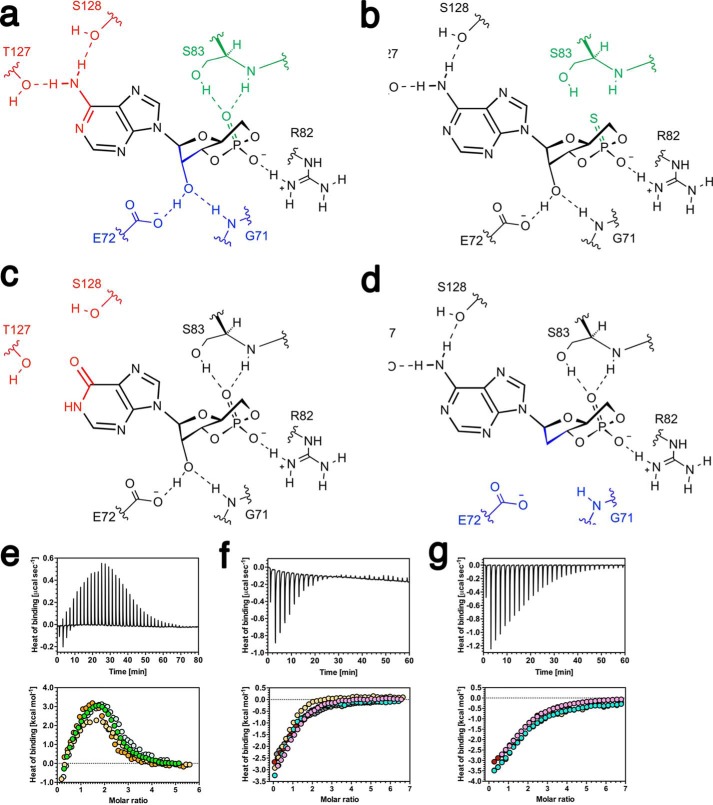
**Disruption of CAP-ligand interactions through the use of cAMP analogs.** *a*, the distribution of binding interactions between cAMP and wild type CAP. *b–d*, binding interactions between Sp-cAMPS (*b*), cIMP (*c*), and 2′-deoxy-cAMP (*d*) and CAP. *e–g*, ITC trace (*upper panel*) and binding isotherm (*lower panel*, the different colored symbols represent individual experiments) for the calorimetric titration of cAMP (*e*), Sp-cAMPS (*f*), and cIMP (*g*) to CAP are shown. The thermodynamic parameters obtained are shown in Table 2.

We used cAMP analogues to weaken CAP-cAMP interactions as an essential test of the predictions of the ENM. Fig. 3*a* shows the bonding interactions between cAMP and CAP to be disrupted through the use of cAMP analogues. This approach to the study of allostery without conformational change has the advantage of requiring only wild type protein, and therefore structural changes between wild type and mutant CAP proteins are eliminated as an experimental variable. We selected Sp-cAMPS to remove the interaction between ligand and the Arg-82/Ser-83 pair (Fig. 3*b*), cIMP to remove the interaction between ligand, and the Thr-127/Ser-128 pair (Fig. 3*c*), and 2′-deoxy-cAMP to remove the interaction between ligand and the Gly-71/Glu-72 pair (Fig. 3*d*). Binding thermodynamics were studied by ITC (Fig. 3, *e–g*). The ITC data were well described by a three-site model, with two major and one minor cAMP binding site (55) and enabled binding parameters (Tables 2 and 3) to be derived. Binding of 2′-deoxy-cAMP to CAP was too weak to be accurately quantified. This observation was confirmed with 2′-NH_2_-cAMP, which also disrupts the interaction between cAMP and Gly-71/Glu-72. This finding is consistent with the demonstration that the bonding interactions between CAP and the 2′-ribose hydroxyl group of cAMP are the major affinity determinant (51, 56). A decrease in positively signed ΔΔ*H* was observed in the binding of Sp-cAMPS and cIMP to CAP when compared with cAMP. Binding of the analogues was also associated with a decrease in the extent of the favorable value of Δ-TΔ*S*. The binding affinities of Sp-cAMPS and cIMP for CAP are therefore reduced in comparison to cAMP, as expected. Furthermore, *K*_2_/*K*_1_ for both analogues is also increased consistent with theoretical predictions (Table 2). The ability to predict the sign of the change in *K*_2_/*K*_1_ is consistent with previous observations (42). The ENM does not incorporate high frequency normal modes of motion. These high frequency modes can couple to the slow modes and are thus involved in the transmission of the allosteric signal, modifying its amplitude. These enslaved fast modes therefore enhance the allosteric free energy change but do not alter the direction of change (57). Because the fast modes are present in the protein (and therefore the experimental data) but not the ENM, the magnitude of the change in *K*_2_/*K*_1_ for the ENM is lower than for the experimental data.

**TABLE 2:** **Allosteric cooperativity from ENM or ITC data** The ratio of the second to first dissociation constants for ligand (*K*_2_/*K*_1_) for wild type CAP protein was calculated from the ENMs or obtained by ITC. The *p* value is for a comparison of means to the wild type for the ITC column (one-way analysis of variance with post hoc Dunnett test).

Ligand	*K*_2_/*K*_1_ (ENM)	Mean *K*_2_/*K*_1_ (ITC)	S.E. (*n*)	*p* value
cAMP	1.17	1.51	0.06 (5)	
Sp-cAMPS ENM^R82/S83^	1.28	10.72	2.79 (6)	<0.05
cIMP ENM^T127/S128^	1.48	9.84	1.99 (4)	<0.05

**TABLE 3:** **Binding parameters for the first and second ligand binding events** The mean values ± S.E. are given for wild type CAP for the first and second ligand binding events. *n* is provided in parentheses. All units are kcal mol^−1^.

Ligand	Δ*H*_1_	Δ*H*_2_	Δ*G*_1_	Δ*G*_2_	−*T*Δ*S*_1_	−*T*Δ*S*_2_
cAMP	−2.2 ± 0.1 (5)	7.6 ± 0.1 (5)	−7.3 ± 0.1 (5)	−7.0 ± 0.1 (5)	−5.1 ± 0.1 (5)	−14.7 ± 0.2 (5)
Sp-cAMPS ENM^R82/S83^	−3.5 ± 0.3 (6)	0.9 ± 0.6 (6)	−6.2 ± 0.1 (6)	−5.1 ± 0.2 (6)	−2.7 ± 0.4 (6)	−5.9 ± 0.5 (6)
cIMP ENM^T127/S128^	−3.8 ± 0.1 (4)	−2.2 ± 0.1 (4)	−6.9 ± 0.0 (4)	−5.9 ± 0.1 (4)	−3.2 ± 0.1 (4)	−3.7 ± 0.1 (4)

**FIGURE 4.**
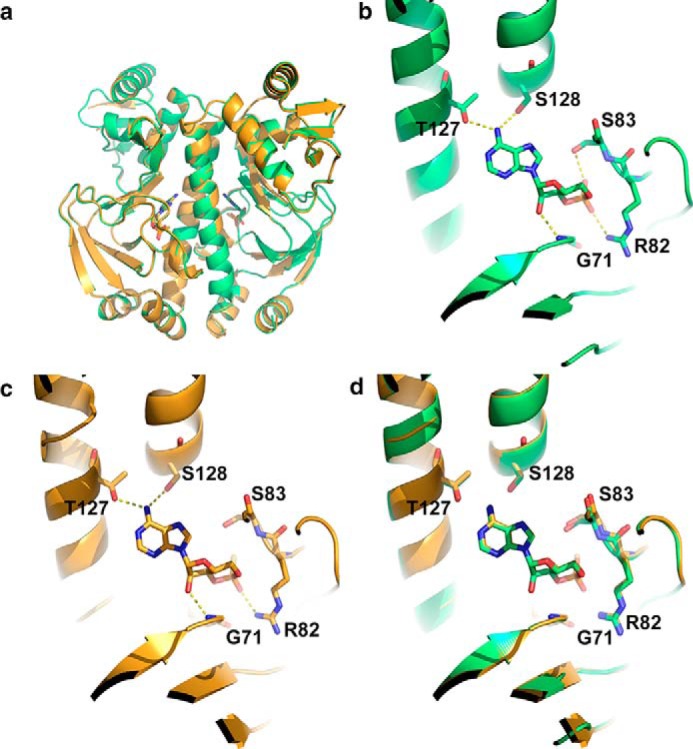
**Analysis of CAP protein structure.** *a*, overlay of the x-ray crystal structures of wild type CAP with cAMP (*green*) and wild type CAP with Sp-cAMPS (*gold*). *b*, close-up of the ligand-binding site of wild type CAP with cAMP showing the hydrogen-bonding network. *c*, close-up of the ligand-binding site of wild type CAP with Sp-cAMPS showing hydrogen-bonding network. *d*, overlay of ligand-binding sites of wild type CAP with cAMP and Sp-cAMPS.

We determined the crystal structure of CAP bound to Sp-cAMPS to confirm that the data were not explained by an unexpected change in the structure of CAP. Fig. 4*a* shows the x-ray crystal structure of CAP bound to Sp-cAMPS overlaid onto the structure of CAP bound to cAMP and demonstrates no significant difference in structure (root mean square deviation = 0.24 Å over 2177 atoms). Fig. 4*b* shows the ligand-binding region of CAP bound to cAMP, and Fig. 4*c* shows the ligand-binding region of CAP bound to Sp-cAMPS. Fig. 4*d* shows the overlay of these two regions. We observe no significant conformational rearrangement of the ligand contacting residues, and the only inferred difference is the loss of a hydrogen bond between the phosphorothioate moiety of Sp-cAMPS and the hydroxyl group of Ser-83. The experimental data are therefore not explained by a change in protein conformation. The computed and experimental data therefore demonstrate an unambiguous relationship between ligand interaction strength and allostery. These data provide a framework within which to investigate how evolution of the protein is constrained by functional requirements for allosteric regulation.

**FIGURE 5.**
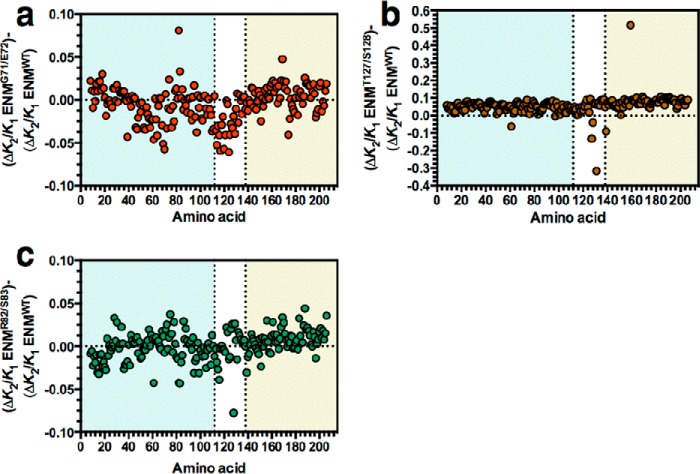
**The influence of CAP-cAMP contacts on changes to allostery induced by second site mutations.** The charts show the change in allosteric cooperativity induced by mutations in ENM^WT^ (Δ*K*_2_/*K*_1_ ENM^WT^) subtracted from the change in allosteric cooperativity induced by the same mutation in ENM^G71/E72^ (Δ*K*_2_/*K*_1_ ENM^G71/E72^) (*a*), ENM^R82/S83^ (Δ*K*_2_/*K*_1_ ENM^R82/S83^) (*b*), or ENM^T127/S128^ (Δ*K*_2_/*K*_1_ ENM^T127/S128^) (*c*) plotted against amino acid number in CAP. The *colored backgrounds* indicate the cAMP-binding domain (*pale blue*), interface forming α helix (*white*), and DNA-binding domain (*red*).

Variant proteins can encompass multiple as well as single mutations. We hypothesized that an analysis of allostery in computed CAP variants with paired mutants could be compared with covarying amino acid pairs in naturally occurring CAP variants. Such an analysis would provide insight into how spatially delocalized residues constrain evolution through an influence on slow relaxation dynamics. We individually reduced the force constants for each Cα in the wild type (ENM^WT^), ENM^G71/E72^, ENM^R82/S83^, or ENM^T127/S128^ models. Fig. 5 (*a–c*) shows the influence of reduced cAMP interaction strength on second site mutations across CAP. ENM^G71/E72^ and ENM^R82/S83^ have varying effects on changes in cooperativity caused by second site mutations. ENM^T127/S128^ has varying effects but more generally tends to increase negative cooperativity. ENM^T127/S128^ has reduced interaction strength across the dimer interface because both monomers contribute to the interaction with cAMP. The increased negative cooperativity is therefore explained by a weakened dimer interface. This has been previously determined as a major contributor to allosteric free energy in a rotational-translational block model (42).

**FIGURE 6.**
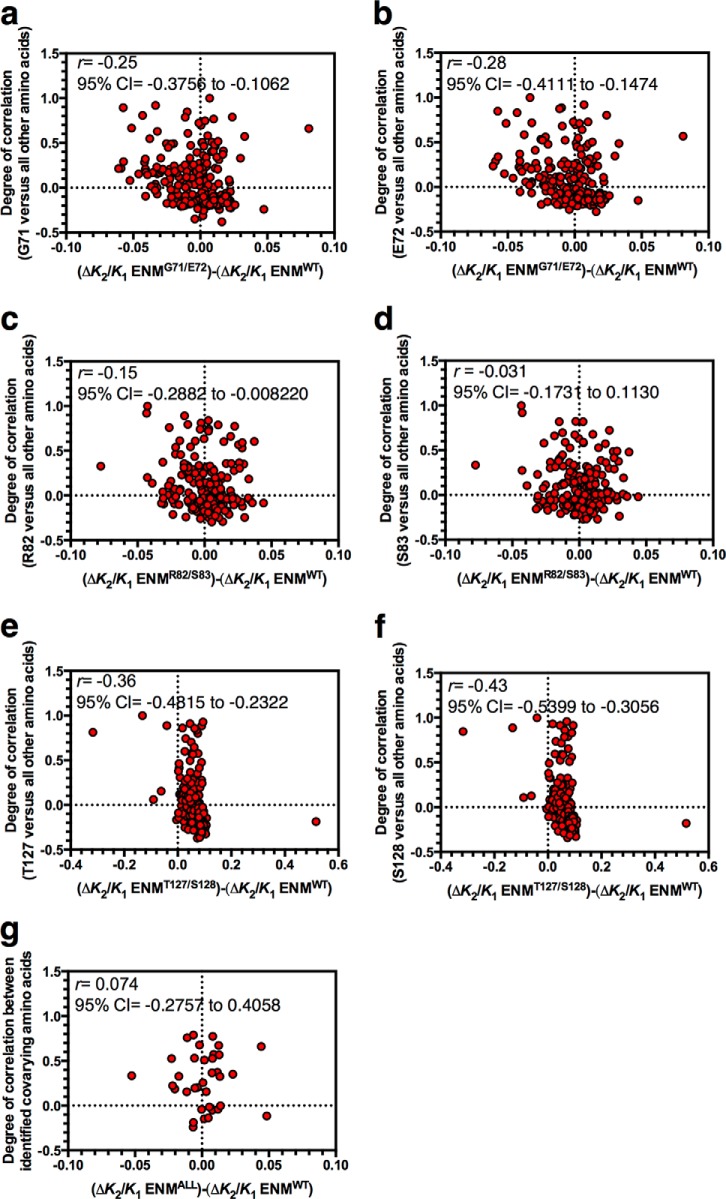
**Correlations between allostery and motion in CAP.** The *y* axes of all charts show the degree of correlated motion between a particular cAMP-contacting residue and every other amino acid in the CAP monomer. The *x* axes of all charts show the change in allosteric cooperativity induced by individual mutation of every amino acid in ENM^WT^ (Δ*K*_2_/*K*_1_ ENM^WT^) subtracted from the change in allosteric cooperativity induced by the same mutation in the ENM with paired mutations in cAMP-contacting residues identified in Fig. 1*D* (ENM named as per Fig. 5). *a*, correlated motion between Gly-71 and all other amino acids plotted against Δ*K*_2_/*K*_1_ ENM^WT^ subtracted from Δ*K*_2_/*K*_1_ ENM^G71/E72^. *b*, correlated motion between Glu-72 and all other amino acids plotted against Δ*K*_2_/*K*_1_ ENM^WT^ subtracted from Δ*K*_2_/*K*_1_ ENM^G71/E72^. *c*, correlated motion between Arg-82 and all other amino acids plotted against Δ*K*_2_/*K*_1_ ENM^WT^ subtracted from Δ*K*_2_/*K*_1_ ENM^R82/S83^. *d*, correlated motion between Ser-83 and all other amino acids plotted against Δ*K*_2_/*K*_1_ ENM^WT^ subtracted from Δ*K*_2_/*K*_1_ ENM^R82/S83^. *e*, correlated motion between Thr-127 and all other amino acids plotted against Δ*K*_2_/*K*_1_ ENM^WT^ subtracted from Δ*K*_2_/*K*_1_ ENM^T127/S128^. *f*, correlated motion between Ser-128 and all other amino acids plotted against Δ*K*_2_/*K*_1_ ENM^WT^ subtracted from Δ*K*_2_/*K*_1_ ENM^T127/S128^. *g*, correlated motion between 35 covarying residue pairs with at least one cAMP-contacting residue from an alignment of CAP variants plotted against Δ*K*_2_/*K*_1_ ENM^WT^ subtracted from Δ*K*_2_/*K*_1_ for the appropriate ENM for that cAMP-contacting residue (Δ*K*_2_/*K*_1_ ENM^ALL^). All analysis has been performed for chain A of the fully cAMP bound ENM.

A prediction of the model for the control of allostery through slow relaxation fluctuations is that allostery occurs as a consequence of correlated motions with amino acid residues (or Cα nodes within the ENM) immediately adjacent to the cAMP binding site. It is a reasonable hypothesis, therefore, that altering such correlated motions through mutation would have an impact upon allostery. More specifically, we predicted that where there is correlated motion between a cAMP-contacting amino acid (***C***) and any other amino acid (***A***), disruption of this correlated motion through mutation of both ***C*** and ***A*** would tend to reduce allosteric cooperativity. This is in contrast to the effect of mutating ***C*** alone, which predominantly increases negative cooperativity (Fig. 1). This effect would occur because mutation of both ***C*** and ***A*** will disrupt the very correlated motions through which slow relaxation fluctuation-driven allostery occurs. To test this hypothesis, we plotted the degree of correlated motion between each cAMP-contacting residue in CAP (every ***C***) and every other amino acid in the same protein chain (***A***) against the influence of mutation at these amino acids on *K*_2_/*K*_1_ (Fig. 6, *a–f*). For example, each data point for Fig. 6*a* represents all amino acids of CAP to be compared against the cAMP-contacting Gly-71 residue. The *y* axis shows the degree of correlated motion between every amino acid (***A***) and Gly-71. The *x* axis shows the effect of mutating each amino acid on allostery (Δ*K*_2_/*K*_1_) in a wild type ENM (ENM^WT^) subtracted from the effect of mutating each amino acid on Δ*K*_2_/*K*_1_ when the strength of cAMP contact (with paired cAMP contacts as per Fig. 1*d*) is reduced at the Gly-71 site (ENM^G71/E72^).

The SRC can provide a measure of the statistical dependence between the plotted nonparametric data of Fig. 6. For five of the six cAMP-contacting residues, we observed a negative value for the SRC coefficient, *r*, between the degree of correlated motion and the effect on allosteric cooperativity. The conclusion drawn from this finding is that where there is a high degree of correlated motion between any amino acid (***A***) and a given cAMP-contacting amino acid (***C***), mutation of ***A*** will drive CAP toward noncooperativity when ***C*** is also mutated. A feature of these correlations and a contributor to the negative value of *r* is the quadrant of negative correlations in motion and increased negative cooperativity (*bottom right quadrants* in Fig. 6, *a–f*). The occupation of this quadrant is a consequence of the significance of global normal modes in carrying the allosteric signal: two distant points in an elastic medium can be anti-correlated in any normal mode of motion, providing they are separated by an odd number of nodes. The physics of entropic modification then adds the tendency toward increased negative cooperativity; the entropic mechanism requires both binding sites to be at anti-nodes of at least one global dynamic mode of the protein. The binding of the first ligand needs to increase the amplitude of the mode by reducing its effective stiffness, whereas the effect of the second is to tighten it.

Natural variation at the cAMP binding site can be rationalized as a requirement for evolving new ligand specificities, fine-tuning cAMP affinity in response to selection pressure, or maintaining cAMP affinity in response to selection at other sites. However, we have shown that altering the cAMP-binding site will alter cooperativity and, when combined with second site mutations, can drive CAP to a nonallosteric variant (Fig. 6, *a–f*). We hypothesized, therefore, that natural CAP variants could provide insight into selection pressures to retain fluctuation-induced allostery in response to changes at the cAMP-binding site. We identified the top 200 covarying amino acids pairs from an alignment of homologous CAP variants. From these, we identified 35 naturally covarying residue pairs with at least one cAMP-contacting residue and tested covariance between the degree of correlated motion and influence on allosteric cooperativity for these pairs. In contrast to the unbiased population analyzed theoretically, no negative correlation was found in the natural set, with a value of *r* of 0.074 indicating a positive but weak association (Fig. 6*g*). A randomization test suggested that the expected theoretical correlation is negative (*r* = −0.23). The observations on the subset of naturally occurring covarying residues are therefore inconsistent with the hypothesis that they are selected randomly from the total theoretical observations of the ENMs (*p* = 0.021). Residues that covary with cAMP-contacting residues therefore permit changes to allosteric cooperativity without allowing the drift in the population to a nonallosteric state that would otherwise occur.

## Discussion

We have demonstrated that the strength of interaction between CAP and cAMP is related to the extent of allosteric cooperativity between cAMP binding sites. As a consequence, if allostery has a selective advantage in CAP, there will be a selection pressure to optimize both ligand affinity and allosteric cooperativity during evolution of the cAMP-binding pocket. Altered interaction strength with cAMP can have varying effects on changes to allostery caused by second site mutations elsewhere in the protein. More generally, we observe a correlation that drives CAP toward a nonallosteric state when altered interaction strength is coupled to second site mutations with which there is a higher degree of correlated motion. This correlation is not observed among naturally occurring covarying residues in CAP, despite our experimental verification of the ENM predictions. This suggests that evolution of the ligand-binding pocket has occurred to permit allosteric tuning without an observable move toward noncooperativity in variant CAP populations.

Some more general conclusions can also be drawn of broad relevance. Fluctuation-induced allosteric cooperativity is predicted to arise naturally as a consequence of an inhomogenous elastic modulus in protein homodimers (44). Evolution of the ligand-binding pocket toward new specificities or in response to new evolutionary challenges must also satisfy other potentially conflicting constraints. These include the accommodation of ligand within the pocket, affinity and specificity for ligand, and structural integrity. We conclude that, where allosteric cooperativity provides a selective advantage, the tuning of fluctuation induced allostery through permissible covarying residues represents a considerable additional constraint on protein evolution. The finding of a relationship between slow relaxation dynamics and protein evolution can be broadly applied beyond allostery. For example, a similar approach could be applied to investigate evolutionary constraints arising from the influence of slow relaxation dynamics on ligand binding.

## Author Contributions

P. D. T. and T. L. R. performed and analyzed the experiments for Tables 2 and 3. T. L. R. and M. J. C. performed and analyzed the experiments for Figs. 1, 2 and 5. P. D. T., L. C. G., and H. J. K performed and analyzed the experiments for Fig. 3. P. D. T. and L. C. G. performed and analyzed the experiments for Fig. 4 and Table 1. T. L. R., M. J. C., L. J. C., and S. A. R performed and analyzed the experiments for Fig. 6. E. P., M. R. W., D. R. W. H., T. C. B. M., and M. J. C. conceived and designed the study. M. J. C. wrote the manuscript. All authors reviewed the results and approved the final version of the manuscript.

## Footnotes

2 The abbreviations used are:

CAP: catabolite activator protein
ENM: elastic network model
ITC: isothermal titration calorimetry
SRC: Spearman's rank order correlation.
